# The COVID-19 Pandemic and Intimate Partner Violence against Women in the Czech Republic: Incidence and Associated Factors

**DOI:** 10.3390/ijerph181910502

**Published:** 2021-10-06

**Authors:** Leona Plášilová, Martin Hůla, Lucie Krejčová, Kateřina Klapilová

**Affiliations:** 1 Faculty of Humanities, Charles University, 182 00 Prague, Czech Republic; lucie.krejcova@nudz.cz (L.K.); katerina.klapilova@nudz.cz (K.K.); 2 Laboratory of Evolutionary Sexology and Psychopathology, Applied Neuroscience and Neuroimaging, National Institute of Mental Health, 250 67 Klecany, Czech Republic; martin.hula@nudz.cz; 3 Faculty of Arts, Charles University, 116 38 Prague, Czech Republic

**Keywords:** COVID-19, pandemic, intimate partner violence, sexual health

## Abstract

Intimate partner violence (IPV) is a burning social issue worldwide. According to global statistics, the incidence of IPV has increased during the COVID-19 pandemic due to restrictive measures (e.g., reduced social contacts, the need to stay at home often with a perpetrator in the same household). This study aims to provide data about the incidence of IPV and its associated factors during the COVID-19 pandemic in the Czech Republic. A representative online sample of 429 Czech women living with a partner at least 3 months before COVID-19 participated in the study. In an online interview, women reported IPV incidents 3 months before and during the first and second waves of the COVID-19 pandemic. Using non-parametric repeated measures ANOVA, a significant difference between the total IPV score and the given time periods was found. In addition, the results of the research showed a significant effect of the tension in the relationship with the partner, depression rate, and partner support on the total IPV score in the first and second wave of the COVID-19 pandemic. These results bring important insights into IPV incidence during the COVID-19 pandemic and suggest factors that might lead to an increased risk of IPV.

## 1. Introduction

With the outbreak of the global COVID-19 pandemic in March 2020, many countries around the world implemented restrictive measures to limit social contact involving restrictions on travel abroad, school closures, restrictions in public places (such as restaurants, clubs, theatres, etc.), the obligation to work from home or the obligation to wear face masks as protection against the spread of infection. Despite the restrictions, nearly 180 million people worldwide have been infected with COVID-19 to date and there have been nearly 3.9 million deaths related to COVID-19 [1].

The impact of the global COVID-19 pandemic is not only health-related but also economic and socioeconomic, as with other major disasters in the past [2]. In this context, the World Health Organization warned at the onset of the pandemic that serious interventions in existing daily routines could result in an increased number of mental health issues for many people [3]. This is also suggested by the study of the U.S. National Institute of Mental Health [4], which said that the number of people reporting symptoms of anxiety, depression or suicidal thoughts had doubled compared to before the pandemic. In addition to healthcare organizations, scientific studies conducted during the pandemic also highlighted the increase in psychopathological and sociopathological phenomena: for example, Lorant et al. [5] pointed to a greater incidence of psychological distress, Taylor et al. [6] found an increase in substance abuse, and Whitehead et al. [7] highlighted the risk of increased poverty due to unemployment caused by the pandemic.

However, one of the most discussed phenomena is the possible increase in intimate partner violence (IPV) during the pandemic. Before the pandemic, one in three women had already experienced physical or sexual violence at least once in their lifetime [8], and the risk of IPV victimization increased even more during the pandemic, according to the WHO report [9]. The closure of schools and the need to work from home meant that people stayed at home with their families for several months, some even longer, mainly for safety reasons. However, as Arenas-Arroyo [10] pointed out, home is not a safe place for everyone. Thus, for some women and children, more time at home also meant more time at home with a perpetrator. Given the existence of different types of violence [11], it is necessary to look at this situation from multiple angles and not focus only on visible violence (i.e., physical violence). The time of the global pandemic may have allowed perpetrators to carry out those types of violence that can be well hidden, such as social (e.g., isolation of the victim from social contacts) or economic violence (see Appendix B for a detailed overview of the incidence of each type of IPV in this study).

In the first weeks and months after the outbreak of the pandemic, crisis lines recorded an increased number of contacts associated with IPV [12,13]. Other indirect evidence of an increase in IPV came from international police statistics [14] or health professionals [15]. Reports about an increase in IPV at the time of the COVID-19 pandemic also came from China, which at that time was one of the most affected countries in the world. According to local police reports, there was a threefold increase in IPV in China [14]. In France, the number of IPV reports increased by 30 percent, and in Brazil, according to available data, reports increased by 40–50 percent [12,16]. This was followed by other studies suggesting an increase in IPV during the pandemic [10,15]; however, the credibility of IPV data during the COVID-19 pandemic may vary depending on the source and sample. In direct relation to the COVID-19 pandemic, Davis et al. [17] in their study found that respondents with COVID-19 infection had an even higher risk of IPV victimization or perpetration.

Given the seriousness of the topic, meta-analysis and systematic review also began to appear relatively quickly [2,18], suggesting that IPV increased during the COVID-19 pandemic. International organizations fighting against IPV have also responded to these findings. United Nations Women [16] released a brief report on the increase in the number of victims of IPV, and 21 world leaders from various international organizations signed a Joint Leaders’ statement pledging to take action to reduce IPV [19].

However, some studies show that there has not always been an increase in IPV cases, e.g., in the study by Jetelina et al. [20], 17% of respondents who screened positive for IPV experienced worsening victimization during the pandemic, but 30% experienced improvement. According to Arenas-Arroyo et al. [10], it is complicated to predict the extent of IPV during a pandemic due to conflicting theories explaining violence as either a form of expressive behavior (that would mean an increase in violence) or as a mode of controlling behavior (that would mean a decrease in violence, as controlling behavior is substituted by restrictive measures). Piquero et al. [18] added that despite the apparent peaks in IPV case numbers in the early weeks of the lockdown, we should be wary of drawing conclusions about violence and the pandemic because the gradual increase in IPV was already occurring in the years prior to the pandemic.

Given the complexity and ambiguity of the whole pandemic situation and its unclear relationship to the IPV phenomenon, it may therefore be difficult to currently interpret existing IPV studies, as UN Women [21] points out in the handbook entitled *Violence Against Women and Girls—Data Collection during COVID-19*. This handbook highlights some of the challenges associated with collecting data on violence against women during a pandemic. For example, it was impossible to use face-to-face contact or some qualitative methods (e.g., focus groups). Furthermore, there was a risk of danger for victims of IPV using online platforms to participate in research, leaving traces in the history of the computer. Some women were not able to access online platforms at all due to the controlling partner. This was also pointed out by Arenas-Arroyo et al. [10], who mention that many studies to date have been based on official records, which may not correspond to reality, as IPV is often an underreported problem; moreover, women’s access to support from justice has become more complicated during the COVID-19 pandemic.

The present study intends to enhance the current knowledge about incidence and factors influencing IPV during pandemic-related restrictions with the data from the Czech Republic (CR)—the central European country that had quite specific development of the pandemic situation. According to the available data [22], the CR experienced the first wave of the COVID-19 pandemic (March to June 2020) with 10,000 cases of COVID-19 infection, similar to other European countries. However, the situation worsened in the autumn with the second wave of the pandemic (September to November 2020), when the country registered 519,752 cases (as of 30 November 2020) and was among the most affected countries in Central Europe. The next severe deterioration came in early 2021, when the CR was ranked as the worst in the world for the number of infections per population [23]. This period (January to March 2021) is often referred to as the third wave of the pandemic, with the country already recording a total of 1,532,834 cases (as of 31 March 2021). As of 15 July 2021, the CR was the 21st most affected country in the world in terms of the number of cases [24]. The CR also has one of the highest rates of IPV in Central Europe—according to the Global Database on Violence against Women [25], about 21% of Czech women will experience physical and/or sexual IPV in their lifetime. The current Czech evidence on IPV during the pandemic shows conflicting results, with police records indicating a decline in the number of expulsions from the common household due to domestic violence over the past year [26], while NGOs note a remarkable increase in cases [27]. However, data based on a representative sample of the population have been missing so far.

The present study therefore aims to compare the self-reported incidence of different types of IPV in the representative sample of Czech women living with a partner before and during the first and second waves of the COVID-19 pandemic. In addition, we decided to investigate the associated factors of IPV during pandemic measures, in the categories of socioeconomic (e.g., age, education, monthly income, number of children) and psychological/interpersonal factors (e.g., overall mental health, perceived tension in the relationship with the partner, perceived tension in the relationship with children, perceived emotional support from partner). The factors were selected based on existing studies that highlight possible associations between IPV and socioeconomic factors such as unemployment during the COVID-19 pandemic [28], changes in financial income [29], or psychological factors such as psychological tension [30]. However, it is important to mention that studies pointing to these factors are sometimes based on a sample of participants from a particular country, and therefore their validity may not be universal but rather country-specific.

The results of this study can serve as a basis for European as well as Czech policymakers or other interested organizations that seek change in the field of violence against women and children and as a basis for establishing an action plan to prevent IPV during similar disasters in the future.

## 2. Materials and Methods

### 2.1. General Study Design

This national study was conducted as part of a broader international I-SHARE (https://ishare.web.unc.edu/, accessed on 5 October 2021) consortium that brings together 33 research institutions from around the world to collect data on sexuality and reproductive health during the COVID-19 pandemic [31]. The questionnaire was developed by the Survey instrument development working group of the I-SHARE consortium. The final form of the questionnaire was discussed and approved by all members of the consortium. Research scales and questionnaires were selected based on topics agreed by the I-SHARE consortium (compliance with COVID-19 social-distancing measures, couple and family relationships, sexual behavior, access to contraceptives, access to reproductive health services, pregnancy and maternal and child health, abortion, sexual and gender-based violence, STIs, mental health, etc.), using gold standard methods that are typically used in sexual health research as well as some newly created questions focused specifically on the COVID-19 pandemic. Each country translated the final questionnaire into its own language and conducted field testing, followed by real data collection. The sampling and timing of questionnaire administration differed from country to country with respect to the development of the pandemic situation. For the purpose of this study, only the part containing the self-report data about IPV from the sample of Czech women were used.

### 2.2. Participants and Sampling Procedure

The Czech participants were recruited via the DataCollect research agency using a computer-assisted web interviewing (CAWI) software. This research agency is a member of the ethical not-for-profit organization ESOMAR and recruits from its own online panel. Respondents were randomly selected by the stratified random sampling with quotas on Czech nationality, region of the CR, size of the place of residence, sex, age, and education. Quotas were determined based on the last census of the Czech Statistical Agency in 2011 [32]. Inclusion criteria for the survey were age (18 years or older), currently residing in the CR and ability to provide online informed consent. The online questionnaire was self-administered and took approximately 27 min. There were 1200 participants in the total sample (612 women, 587 men, 1 other) with a response rate of 86,1%. From the total sample, only women who had a partner at least 3 months before the onset of the pandemic were subsequently filtered for the purposes of this study.

Before completing the questionnaire, the participants were acquainted with the objectives of the study and confirmed that they understood the informed consent and the conditions of participation by pressing the “agree” button. They were also informed that it would not be possible to link their answers to a specific person and that we guaranteed anonymity (using only a randomly generated ID) and data protection. Participation in the study was voluntary, and respondents could also stop completing the questionnaire at any time if they wished. They were contacted based on a quota selection from the research agency’s panel, by e-mail invitation or notification in the mobile application. The rewards for participation were points that could be exchanged in the research agency’s system for money, vouchers, goods, or charity donations.

**Figure 1:**
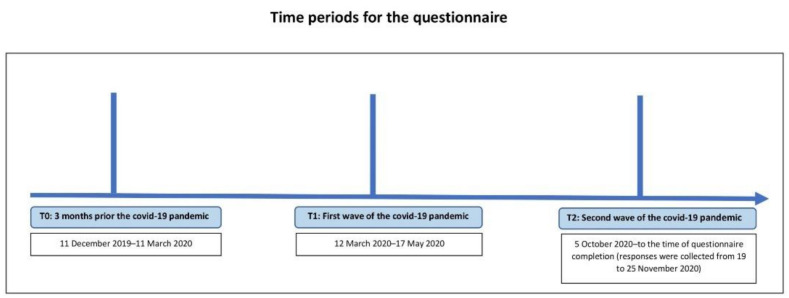
Time periods for the questionnaire.

Responses were collected from 19 to 25 November 2020. Participants were clearly informed about the specific time periods we were asking about (see Figure 1): (1) three months before the COVID-19 pandemic (t0); (2) the first wave of the COVID-19 pandemic: 12 March 2020 to 17 May 2020 (t1); (3) the second wave of the COVID-19 pandemic: 5 October 2020 to the time of questionnaire completion (t2). They were asked to adjust their retrospective self-reported answers to these specific periods framed by the concrete dates that were characterized by strong restrictive measures in the CR.

### 2.3. Instruments

As this research study focuses specifically on the topic of IPV, only this part of the questionnaire will be described. The whole questionnaire with all sections is available on request.

The sociodemographic variables used in this study were: sex, age, education, monthly income, change in monthly income during the first wave of COVID-19 pandemic, change in monthly income during the second wave of COVID-19 pandemic, number of children, items related to relationship status, region of the CR, size of the place of residence. Questions related to sociodemography were mandatory.

The dependent variable was an experience with different types of violence from an intimate partner measured by an adapted shortened six-item questionnaire derived from the WHO IPV interview (see Appendix A), which is widely used in research focusing on IPV [33]. This questionnaire covers a total of six different types of IPV: economic, social, emotional, physical, sexual–psychological, sexual–physical. The last two types of sexual violence differ as follows: sexual–psychological violence occurs when the perpetrator puts psychological pressure on the victim to have sexual intercourse with him, while sexual–physical violence occurs when the perpetrator physically forces the victim to have sexual intercourse with him. Respondents were asked about their experience with six types of violence in the 3 times of data sampling: t0, t1 and t2. The answer options were: I had no experience with this type of violence in that time period/Yes, I experienced it once in that time period/Yes, I experienced it several times in that time period. For the purpose of this study, we calculated the total IPV score by adding the values of all six items. Each item ranged from 0 (no experience) to 2 (experienced several times), resulting in a minimal total score 0 and maximal total score 12. Questions related to IPV were not mandatory.

The independent variables (IV) were divided into two categories: socioeconomic and psychological/interpersonal. Socioeconomic IV included some variables from the sociodemographic part of the questionnaire (age, education, monthly income, change in monthly income during first/second wave of COVID-19 pandemic, number of children). Psychological/interpersonal IV were not mandatory and included the following:

- An overall mental health (t1, t2), assessed by the question “How would you rate your overall mental health?” with answer options: Poor/Fair/Good/Very good/Excellent.
- A depression rate (t0, t1, t2) assessed by the question “Have you been feeling down and depressed more or less since the start of the lockdown?” with answer options: A lot more/More/About the same/Less/A lot less. This question was taken from the standardized questionnaire for screening for depressive symptoms PRIME-MD [34].
- An emotional support provided by the partner (t0), assessed by the question: “In the three months before the COVID-19 social distancing measures, how much would you say your partner provided you with emotional support?” with answer options: A lot/Some support/Little support/No support.
- A tension in the relationship with the partner (t0), assessed by the question: “In the three months before the COVID-19 social distancing measures, how often did you experience tension in your relationship to your partner/spouse?” with answer options: Never/Monthly or less/2–4 times a month/2–3 times a week/4 or more times a week.
- A tension in the relationship with one’s own children (t0), assessed by the question: “In the three months before the COVID-19 social distancing measures, how often did you experience tension in your relationship to your children? (only for those living with children)” with answer options: Never/Monthly or less/2–4 times a month/2–3 times a week/4 or more times a week.

### 2.4. Analytical Strategy

We used R 4.0.3 [35] and RStudio 1.3.1093 [36] (R Foundation for Statistical Computing, Vienna, Austria) for the statistical analyses. We set the alpha level for statistical tests to 0.05.

To test whether there were any differences between the total IPV scores across time periods (t0, t1, t2), we used repeated measures ANOVA, where the total IPV score was the dependent variable, the time period was the factor (with three levels: t0, t1, t2) and the respondent’s ID represented the blocking variable. Visual check of the IPV score distributions in each time period revealed that the scores were clearly non-normally distributed. For this reason, we used non-parametric Friedman’s ANOVA. To test the differences between groups, we used paired Wilcoxon signed-rank tests and estimated the effect size by r (calculated as r = Z/sqrt(N), where Z is the test statistic and N is the total number of pairs).

We were interested in the possible effects of the demographic and other above-mentioned independent variables on the total IPV score during the first and second pandemic wave. To see which variables might have an effect, we first created a matrix of Spearman’s correlations (see Supplementary Table S1). We then ran a linear regression model, where the total IPV score in t1 served as the dependent variable, and the variables from the matrix that correlated with the total IPV score in the t1 (rho higher than 0.25 or lower than −0.25) served as independent variables. If there was a strong correlation between the independent variables (rho higher than 0.7 or lower than −0.7), we included only one of them in the model. We used the same approach for a second model, where the total IPV in the t2 was the dependent variable.

### 2.5. Ethics Consideration

This study was approved by the Ethics Committee of the National Institute of Mental Health in Prague, Czech Republic (No. 149/20).

## 3. Results

The final sample consisted of 429 women (age range: 18–84 years, M age = 48.45; SD = 16.25); 3.96% of respondents completed primary school, 7.46% of respondents had some years of secondary school, 64.57% of respondents completed secondary school, 3.73% of respondents had some time in college/university, and 17.94% of respondents completed college/university (see Table 1). None of the participants chose the option “No formal education” or “Some primary school”.

For a descriptive analysis of IPV scores in given time periods, see Table 2. Friedman’s ANOVA found a significant difference between the total IPV score and the time period (chi-squared = 25.04, df = 2, *p*-value < 0.0001). The subsequent paired Wilcoxon sum-rank tests revealed that there was a significant difference between the t0 period and both the t1 (V = 756, *p*-value = 0.018, 95% C.I. of the score difference = 0.50–1.50 points, r = 0.18, 95% C.I. of r = 0.09–0.25) and t2 (V = 1217.5, *p*-value = 0.00056, 95% C.I. of the score difference = 1.00–2.00 points, r = 0.22, 95% C.I. of r = 0.13–0.29). There was no difference in the total IPV score between the t1 and t2 (V = 247.5, *p*-value = 0.17, 95% C.I. of the score difference = −1.50–0.00026 points, r = 0.083, 95% C.I. of r = 0.0082–0.18). Both significant differences had small effect sizes with differences of 1–1.5 points out of the theoretical maximum of 12 points.

We ran a regression model, where the total IPV score during the first pandemic wave served as the dependent variable. Only complete observations were used for the regression model. The final dataset consisted of 390 participants (39 incomplete). Based on the correlation matrix, we included tension with the partner before the pandemic and support from the partner before the pandemic as predictors. Depression rates in t0, t1 and t2 also correlated with the total IPV score. However, they also very strongly correlated with each other. Therefore, we decided to include only the depression rate in t1. Other variables did not correlate with the total IPV score in t1. The model (F3,399 = 29.37, *p*-value: < 0.0001, adj R2 = 0.17) revealed a significant positive effect of the tension in the relationship with the partner in t0 and depression in t1, and a significant negative effect of partner support in t0 on the total IPV score (see Table 3).

We repeated this procedure with the total IPV score in t2. Again, the only variables that sufficiently correlated with the total IPV score were the tension in the relationship with the partner in t0, partner support in t0, and the depression rate in t2. The model (F3,404 = 23.26, *p*-value < 0.0001, adj R2 = 0.14) showed a significant positive effect of the tension in the relationship with the partner in t0 and negative effect of the partner support in t0. This time, the depression rate in t2 was not significant (see Table 4).

## 4. Discussion

The aim of this study was to determine the incidence of IPV in Czech women during the first and second waves of the COVID-19 pandemic and to compare the incidence of IPV in these periods with the period before the COVID-19 pandemic. To the best of our knowledge, there are no studies yet to compare data on IPV in different waves of the COVID-19 pandemic. A significant difference was observed in the incidence of IPV in the first wave of the COVID-19 pandemic compared to the pre-pandemic period, as well as in the second wave compared to the pre-pandemic period. A lower incidence of IPV was found in both waves of the COVID-19 pandemic compared to the pre-pandemic period. However, in both cases, the effect sizes were small. No significant difference in the incidence of IPV was observed when comparing the first and second waves of the COVID-19 pandemic.

Lyons and Brewer [37] point to several mechanisms that may directly or indirectly increase the risk of IPV during global pandemics, such as financial difficulties of households, social isolation, and limited availability of services for IPV victims. While social isolation during the COVID-19 pandemic in the CR may have had an impact on the population and their daily lives [38], unemployment (associated with financial situation of households) has increased only slightly [39]. This may be due to extensive support provided by government programs to employers to maintain jobs during the COVID-19 pandemic. Maintaining household income during the COVID-19 pandemic could thus indirectly act as a preventive factor for the occurrence of IPV. Another mechanism mentioned by Lyons and Brewer [37], the limited availability of services for IPV victims, was only a partial problem in the CR, especially at the beginning of the pandemic, when most institutions and organizations were paralyzed by the pandemic situation. However, in the first months after the outbreak of the COVID-19 pandemic, these services began to work again using alternative means of communication such as online chats, video calls or telephone crisis lines, often with extended opening hours. According to a survey made by the Association of Intervention Centers (focused on helping victims of domestic violence) operating in the CR, as many as 90% of respondents using help from IPV organizations in the first wave of the COVID-19 pandemic considered the offer of services to be sufficient [40]. These underlying mechanisms, such as government intervention to support employment and maintaining the availability of services to help IPV victims during the COVID-19 pandemic, may explain why a significant difference in the incidence of IPV was observed, but with little practical significance given the acquired effect size. However, this does not mean that the importance of fighting against IPV during the COVID-19 pandemic should be underestimated. Given that the COVID-19 pandemic has affected almost every country in the world, and that each country has had a different approach to the situation, it is necessary to examine this phenomenon while considering the course of the COVID-19 pandemic in particular countries and restrictions put in place, and also national cultural specificities such as community attitudes to violence or functional networks of victim support services. Similarly to Krishnamurti et al. [41], who also did not observe the expected increase in IPV during the COVID-19 pandemic in their study, it is important to note that the lack of evidence for the presence of IPV does not necessarily mean that it does not occur. Due to the sensitivity of this topic, it is necessary to be aware of the situation of victims, who often do not have a chance to participate in research at all, because their conduct may be controlled by the perpetrator.

Furthermore, we examined the risk factors associated with the incidence of IPV, in two categories: sociodemographic (age, education, monthly income, change in monthly income during the first wave of the COVID-19 pandemic, change in monthly income during the second wave of the COVID-19 pandemic, number of children) and psychological/interpersonal variables (overall mental health during the first/second wave of the COVID-19 pandemic, depression rate 3 months prior to the COVID-19 pandemic and during the first/second wave of the COVID-19 pandemic, tension in the relationship with the partner 3 months prior to the COVID-19 pandemic, tension in the relationship with children 3 months prior to the COVID-19 pandemic and emotional support from the partner 3 months prior to the COVID-19 pandemic. Based on the correlation matrix, we included the tension with the partner 3 months prior to the COVID-19 pandemic, the emotional support from the partner 3 months prior to the COVID-19 pandemic, and the depression rate in the regression model for the first and second waves of the COVID-19 pandemic, respectively. All these variables were significant for the first wave of the COVID-19 pandemic, while for the second wave of the COVID-19 pandemic the depression rate did not show significance. One of the reasons for this may be a better knowledge of the whole situation around the COVID-19 pandemic at the time of the second wave. Sufficient information can reduce uncertainty in crisis situations and thus prevent mental health deterioration.

Similarly to Gresham et al. [42], whose study showed that IPV victimization during the COVID-19 pandemic was not associated with poorer mental health, this study also did not show that mental health was associated with this phenomenon. However, the depression rate, which may be one of the symptoms of poorer mental health, was partially shown to be related to the incidence of IPV in this study (in the model for the first wave of the COVID-19 pandemic but not in the model for the second wave). Importantly, these data do not indicate causality; however, there is growing evidence that there is a relationship between IPV and depression [43].

In addition to mental health, tension in the relationship with the partner and emotional support from the partner were further examined as psychological/interpersonal factors. These factors showed significance (the former positive and the latter negative) for the regression models for both waves of the COVID-19 pandemic. The tension in the relationship with the partner and the emotional support provided by the partner can relate to the overall satisfaction with the relationship [44]. Existing studies suggest that relationship satisfaction and IPV are negatively associated [45,46], which intuitively fits the results of our study. However, relationship satisfaction is a complex phenomenon and its relationship to the risk of IPV needs to be further explored.

An associated sociodemographic risk factor for IPV is having children, as some studies show. For example, Acavedo et al. [47] pointed out that participants with two or more children are at more than twice the risk of IPV than those with only one child. As the authors mentioned, children can be one of the reasons for victims to maintain a relationship with the perpetrator. Furthermore, childcare can be associated with psychological, financial, and other life stressors that affect mainly women as primary caregivers. In our study, it was surprisingly not shown that the number of children or tension in the relationship with the children would be associated with the incidence of IPV during the COVID-19 pandemic, even though there were enough women with children in the sample (the mean number of children per participant was 2). This may be due to a large variance in the age of the participants (M = 48 years, SD = 15), of which more than half were older than 45 years. This could mean that their children can potentially live separately and do not require primary childcare resulting in caregiver stress, which can eventually be indirectly reflected in higher IPV [48] as mentioned above.

Another sociodemographic factor related to the incidence of IPV is younger age [49] and lower income [50], and in the time of the pandemic specifically a change in income [29]. We tested both of these factors and neither showed significance. Although younger age is generally a frequently mentioned risk factor for the incidence of IPV, it has not yet been shown to be significant in the context of studies on the incidence of IPV during the COVID-19 pandemic. This may be due to the different coping mechanisms that young people use during the COVID-19 pandemic [51], or due to the changes of life values during the global pandemic [52]. These changes may be reflected in overall lifestyle, which in some cases may become more mature in young people and, as a result, less vulnerable to IPV. In contrast, loss of income is widely mentioned as one of the factors increasing the risk of IPV during the COVID-19 pandemic. However, four-fifths of the women in our sample did not experience any change in income during the pandemic, which may be due to, as already indicated, government funds to support employment during the COVID-19 pandemic. Despite the importance of this factor for predicting the incidence of IPV in other countries, it turns out that it may not be suitable for the pandemic situation in the CR.

Although this study is based on a sample with adequate predictive value derived from a representative sample, it also has several limitations. Firstly, due to the online data collection, participants were required to have access to the Internet, which may cause selection bias, especially due to the sensitivity of the topic. IPV victims often do not have access to a computer, are strictly controlled by the perpetrator or do not feel safe answering questions about violence. In this research, the protection of the participant and the traces in Internet history were partially solved by the neutral name of the survey website (“On-line survey”). Secondly, because the questionnaire was based on self-reporting, it is possible that some responses may be influenced by social desirability, as it sometimes happens in IPV research [53]. Thirdly, the participants were interviewed retrospectively for the period before the COVID-19 pandemic and for the first wave of the COVID-19 pandemic, which may cause bias in their responses due to inaccurate memories. Finally, only women who had a partner for at least 3 months before the start of the COVID-19 pandemic were selected as participants. However, other important factors such as the length of the relationship or living in the same household were not considered and could potentially also play a role in the incidence of IPV during the COVID-19 pandemic. Fourthly, the options of the questionnaire were limited to only three (never, once, several times). This can be limiting for participants who experience IPV more often than “several times”, e.g., on a daily basis. Another limit is the statistical procedure based on the analysis of the correlation matrix. If there was a strong correlation of variables (rho higher than 0.7 or lower than −0.7) we included only one of the variables, which can cause a suppression effect (false improvement of the prediction of the dependent variable due to the inclusion of an independent variable that does not originally correlate with the dependent variable).

Finally, it is important to mention that IPV does not only concern women, but also men. To date, there is little research on IPV in men as victims. Thus, it is a challenge for future research.

## 5. Conclusions

In conclusion, this study suggests that there is a significant difference in the incidence of IPV at 3 months prior to the COVID-19 pandemic compared to the first and second wave of the COVID-19 pandemic, however with a small effect size. Factors indicating an association with IPV in this study are emotional support from the partner at 3 months prior to the pandemic, tension in the relationship with the partner at 3 months prior to the pandemic, and partly also depression rate of the victim. These results are important for filling the gap in knowledge about the incidence of IPV during the COVID-19 pandemic in the CR, which has not yet received much attention in the scientific field. This study also offers a unique comparison of the incidence of IPV between the first and second waves of the COVID-19 pandemic, which is important for capturing the progression of this phenomenon. Still, the mechanism of progression of IPV during long-term global disasters such as this needs to be further explored.

Future research should focus on a longitudinal investigation of the relationship between the incidence of the COVID-19 pandemic and IPV and possible mediators of their relationship. It can be useful to compare the incidence of IPV in different phases of the COVID-19 pandemic in different countries and with different restrictive measures. Then it will be possible to conclude which measures may be the greatest risk to the incidence of IPV, and therefore policymakers should take them into account when planning COVID-19 pandemic management strategies.

## Figures and Tables

**Table 1:** Descriptive analysis of sociodemographic variables.

Variable	Categories	N (%)	Mean (SD)	Mode
Age	18–24	26 (6.06%)		35–44
	25–34	79 (18.41%)		
	35–44	97 (22.61%)	48.45 (16.25)	
	45–54	76 (17.72%)		
	55–64	57 (13.28%)		
	65<	94 (21.91%)		
Number of children	0	75 (17.48%)		
	1	104 (24.24%)		
	2	170 (39.62%)		
	3	61 (14.22%)	1.51 (1.19)	2.00
	4	15 (3.50%)		
	5	4 (0.93%)		
Monthly income	N/A	44 (10.25%)		
	1	19 (4.43%)		
	2	18 (4.19%)		
	3	38 (8.86%)		
	4	108 (25.17%)		4.00
	5	100 (23.31%)		
	6	74 (17.25%)		
	7	24 (5.60%)		
	8	3 (0.70%)		
	9	1 (0.23%)		
	Completed primary school	17 (3.96%)		
Education	Some secondary school	32(7.46%)		Completed secondary school
	Completed secondary school	277 (64.57%)		
	Some college/university	16 (3.73%)		
	Completed college/university	77 (17.94%)		
	Other	10 (2.33%)		

**Table 2:** Descriptive analysis of total IPV scores ^1^ in given time periods ^2^.

	Median	Mean	SD	Min	Max	Skewness	Kurtosis
t0	0.00	0.617	1.663	0.00	10.00	3.378	11.722
t1	0.00	0.519	1.670	0.00	12.00	4.170	18.760
t2	0.00	0.466	1.601	0.00	12.00	4.553	22.933

^1^ score range 0–12. ^2^ t0 = 3 months prior the COVID-19 pandemic, t1 = first wave of COVID-19 pandemic, t2 = second wave of COVID-19 pandemic.

**Table 3:** Coefficient estimates of the regression model. The total IPV score during the t1 served as the dependent variable.

	CoefficientsEstimate	Std Error	t	*p*	Lower 95% CI	Upper 95% CI
Intercept	0.652	0.385	1.693	0.0912	−0.105	1.408
Tension in the relationship with the partner (t0)	0.509	0.0883	5.762	<0.0001	0.335	0.682
Partner support (t0)	−0.317	0. 0938	−3.382	0.00079	−0.502	−0.133
Depression rate (t1)	0.228	0.103	2.202	0.0282	0.0244	0.431

**Table 4:** Coefficient estimates of the regression model. The total IPV score during the t2 served as the dependent variable.

	CoefficientsEstimate	Std Error	t	*p*	Lower 95% CI	Upper 95% CI
Intercept	0.787	0.367	2.145	0.0325	0.0659	1.508
Tension in the relationship with the partner (t0)	0.428	0.0849	5.041	<0.0001	0.261	0.595
Partner support (t0)	−0.316	0. 0904	−3.495	0.000527	−0.494	−0.138
Depression rate (t2)	0.157	0.0932	1.684	0.0930	−0.0263	0.340

## Data Availability

The data presented in this study are available on request from the corresponding author. The data are not publicly available due to privacy.

## Supplementary Materials

The following are available online at https://www.mdpi.com/article/10.3390/ijerph181910502/s1, Table S1: Correlation matrix of key variables.

## Appendix A

Adapted shortened six-item questionnaire derived from the WHO IPV interview (35).

For each of the time periods (t0, t1, t2), please indicate if you have had experience with the following:

1. Social violence: Has your partner tried to restrict (online or phone) contact with your family?
2. Emotional violence: Has your partner insulted you or made you feel bad about yourself?
3. Financial violence: Has your partner ever not provided money to run the house or look after the children, while having money for other things?
4. Physical violence: Has your partner slapped, pushed, hit, kicked or choked you or thrown something at you that could hurt you?
5. Sexual violence (physical force): Has your partner physically forced you to have sexual intercourse when you did not want to?
6. Sexual violence (psychological force): Has your partner made you have sexual intercourse when you did not want to because you were afraid of what your partner might do?

## Appendix B

**Table A1:** Overview of the incidence of individual types of IPV in three time periods.

Type of Violence	3 Months Prior				First Wave				Second Wave			
	N/A	No	Once	Several Times	N/A	No	Once	Several Times	N/A	No	Once	Several Times
Social	11 (2.6%)	406 (94.6%)	7 (1.6%)	5 (1.2%)	7 (1.6%)	410 (95.6%)	4 (0.9%)	8 (1.9%)	7 (1.6%)	411 (95.8%)	4 (0.9%)	7 (1.6%)
Emotional	12 (2.8%)	373 (86.9%)	13 (3.0%)	31 (7.2%)	10 (2.3%)	378 (88.1%)	13 (3.0%)	28 (6.5%)	8 (1.9%)	385 (89.7%)	11 (2.6%)	25 (5.8%)
Financial	23 (5.4%)	374 (87.2%)	15 (3.5%)	17 (4.0%)	14 (3.3%)	388 (90.4%)	10 (2.3%)	17 (4.0%)	17 (4.0%)	386 (90.0%)	9 (2.1%)	17 (4.0%)
Physical	12 (2.8%)	392 (91.4%)	16 (3.7%)	9 (2.1%)	10 (2.3%)	403 (93.9%)	7 (1.6%)	9 (2.1%)	10 (2.3%)	405 (94.4%)	5 (1.2%)	9 (2.1%)
Sexual (physical force)	10 (2.3%)	378 (88.1%)	18 (4.2%)	23 (5.4%)	11 (2.6%)	392 (91.4%)	11 (2.6%)	15 (3.5%)	10 (2.3%)	400 (93.2%)	8 (1.9)	11 (2.6%)
Sexual (psychological force)	10 (2.3%)	402 (93.7%)	8 (1.9%)	9 (2.1%)	11 (2.6%)	404 (94.2%)	3 (0.7%)	11 (2.6%)	10 (2.3%)	408 (95.1%)	3 (0.7%)	8 (1.9%)
