# Supplementary material for: The COVID-19 Pandemic and Intimate Partner Violence against Women in the Czech Republic: Incidence and Associated Factors
Source: Int J Environ Res Public Health. 2021 Oct 6;18(19):10502. doi: 10.3390/ijerph181910502 (PMC8508297; doi:10.3390/ijerph181910502)
Supplement: Supplementary file 1 [file ijerph-18-10502-s001.zip › ijerph-1321973-supplementary.pdf]

Supplementary Table. S1 Correlation matrix of key variables.

	1	2	3	4	5	6	7	8	9	10	11	12	13	14	15	16	17
1. IPV score (t0)	1.000																
2. IPV score (t1)	0.847***	1.000															
3. IPV score (t2)	0.780***	0.839***	1.000														
4. Tension in relationship with a partner (t0)	0.364***	0.356***	0.341***	1.000													
5. Tension in relationship with children (t0)	0.244***	0.190***	0.185***	0.479***	1.000												
6. Partner support (t0)	-0.300***	-0.290***	-0.268***	-0.315***	-0.159**	1.000											
7. Number of children	-0.064	-0.054	-0.028	-0.006	0.079	-0.014	1.000										
8. Schooling	0.045	0.031	0.026	0.053	0.138*	0.062	-0.063	1.000									
9. Net income	-0.002	0.023	0.042	0.036	0.148**	0.031	0.016	0.294***	1.000								
10. Age	-0.152**	-0.183***	-0.177***	-0.221***	-0.228***	0.008	0.324***	-0.098*	-0.039	1.000							
11. Income change (t1)	-0.131**	-0.079	-0.002	-0.107*	-0.081	0.104*	-0.005	0.114*	0.119*	0.141**	1.000						
12. Income change (t2)	-0.127*	-0.069	-0.037	-0.143**	-0.113*	0.123*	-0.005	0.093	0.126*	0.079	0.674***	1.000					
13. Depression rate (t0)	0.298***	0.247***	0.273***	0.373***	0.244***	-0.212***	-0.089	-0.030	-0.053	-0.208***	-0.137**	-0.193***	1.000				
14. Depression rate (t1)	0.300***	0.254***	0.277***	0.381***	0.295***	-0.162***	-0.089	0.047	-0.057	-0.269***	-0.174***	-0.191***	0.767***	1.000			
15. Depression rate (t2)	0.270***	0.232***	0.251***	0.374***	0.286***	-0.147**	-0.128**	0.038	-0.040	-0.236***	-0.160***	-0.237***	0.707***	0.808***	1.000		
16. Mental health (t1)	-0.226***	-0.177***	-0.217***	-0.196***	-0.196***	0.157**	0.078	0.105*	0.161**	0.178***	0.146**	0.139**	-0.453***	-0.560***	-0.446***	1.000	
17. Mental health (t2)	-0.218***	-0.146**	-0.209***	-0.202***	-0.168**	0.203***	0.108*	0.138**	0.115*	0.140**	0.143**	0.163***	-0.475***	-0.522***	-0.559***	0.841***	1.000

*p<0.05; **p<0.01;***p<0.01
